# Magnified Sediment Export of Small Mountainous Rivers in Taiwan: Chain Reactions from Increased Rainfall Intensity under Global Warming

**DOI:** 10.1371/journal.pone.0138283

**Published:** 2015-09-15

**Authors:** Tsung-Yu Lee, Jr-Chuan Huang, Jun-Yi Lee, Shih-Hao Jien, Franz Zehetner, Shuh-Ji Kao

**Affiliations:** 1 Department of Geography, National Taiwan Normal University, Taipei, Taiwan; 2 Department of Geography, National Taiwan University, Taipei, Taiwan; 3 Department of Soil and Water Conservation, National Pingtung University of Science and Technology, Pingtung, Taiwan; 4 Institute of Soil Research, University of Natural Resources and Life Sciences, Vienna, Austria; 5 State Key Laboratory of Marine Environmental Science, Xiamen University, Xiamen, China; NSYSU, TAIWAN

## Abstract

Fluvial sediment export from small mountainous rivers in Oceania has global biogeochemical significance affecting the turnover rate and export of terrestrial carbon, which might be speeding up at the recognized conditions of increased rainfall intensity. In this study, the historical runoff and sediment export from 16 major rivers in Taiwan are investigated and separated into an early stage (1970–1989) and a recent stage (1990–2010) to illustrate the changes of both runoff and sediment export. The mean daily sediment export from Taiwan Island in the recent stage significantly increased by >80% with subtle increase in daily runoff, indicating more sediment being delivered to the ocean per unit of runoff in the recent stage. The medians of the runoff depth and sediment yield extremes (99.0–99.9 percentiles) among the 16 rivers increased by 6.5%-37% and 62%-94%, respectively, reflecting the disproportionately magnified response of sediment export to the increased runoff. Taiwan is facing increasing event rainfall intensity which has resulted in chain reactions on magnified runoff and sediment export responses. As the globe is warming, rainfall extremes, which are proved to be temperature-dependent, very likely intensify runoff and trigger more sediment associated hazards. Such impacts might occur globally because significant increases of high-intensity precipitation have been observed not only in Taiwan but over most land areas of the globe.

## Introduction

The small mountainous rivers (SMRs) in Oceania are one of the well-known hot spots of global sediment export from land to ocean; they drain only ~2.5% of the global land surface but collectively transport ~40% of the annual global land-to-ocean sediment export [1]. Fluvial sediment export is tightly related to the evolution of geomorphology [2–3], off-shore aquatic ecosystems [4–5], physical and chemical weathering [6–7], and even the occurrence of earthquakes [8]. Recently, the scientific community has paid much more attention to it because the fate of eroded sediment is involved in the global carbon cycle, e.g. through the exhumation of carbon stored in the bedrock [9–10], fate of terrestrial biogenic organic carbon [11–12] and sequestration of carbon from different sources [13–16]. Among them, the terrestrial biogenic organic carbon, i.e. the recently-fixed carbon dioxide from the atmosphere, is of particular interest [17]. More than one third of the organic carbon delivered to the oceans via erosion and riverine transport comes from sediment-laden rivers that drain the mountains in the western Pacific region where tropical cyclones invade frequently [18–19]. A small change in SMRs might significantly alter the global sediment budget and associated consequences. Although fluvial sediment export has high global significance, the opportunities to analyze long-term sediment export data from SMRs are scarce owing to inadequate measurements. Taiwan, as one of the most representative islands in Oceania, has relatively good hydrometric records to examine the history of fluvial sediment export.

In Taiwan, landslides are the major source of fluvial sediment export [20]. Intense rainfall combined with high tectonic rates drive rapid mass wasting and fluvial sediment transfer [3]. These processes might be enhanced by increasing rainfall intensity [21] to which landslide amount is directly related [22–23]. Previous studies demonstrated that the rainfall extremes in China, Japan, India and Taiwan have increased by ~90% [21], and have resulted in an increase of >100% in runoff extremes [24], which act as the major carrier of fluvial sediment [25]. Given the increased rainfall intensity and magnified runoff response [24], we are inspired to explore if there are temporal trends discernible for sediment export by investigating the historical records from Taiwan. This is particularly crucial because most of the catastrophic events in Taiwan are associated with landslides and the resulted sediment transport. It is worthwhile to note that significant increases of high-intensity precipitation have been observed not only in Taiwan but over most of the land areas of the globe in the last few decades [26–28].

**Fig 1:**
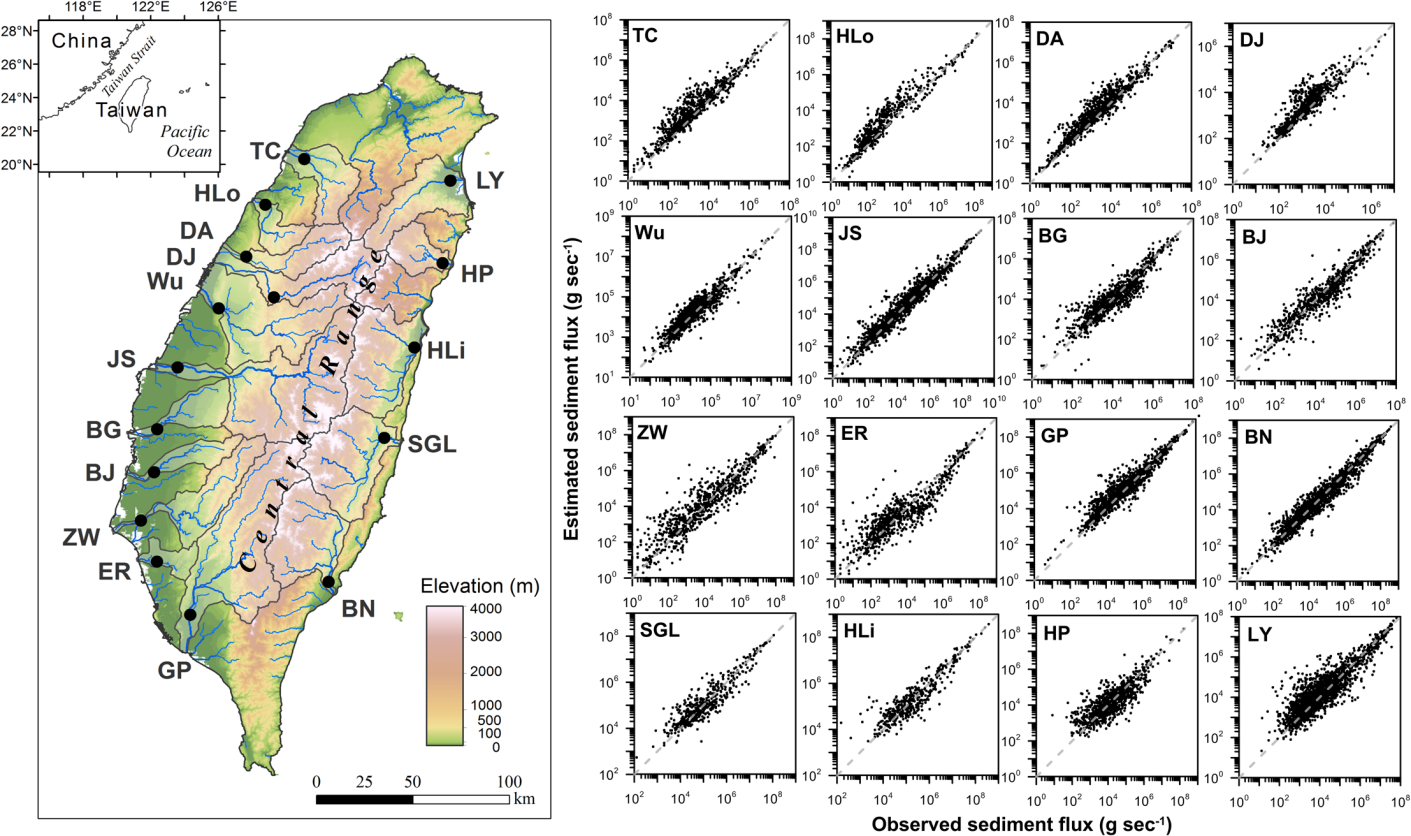
The locations of Taiwan and the hydrometric gauges for the 16 primary rivers investigated in this study shown on the topography map (left panel), and scatter plots of estimated against observed sediment fluxes for the 16 rivers (right panels). The 1:1 lines are also illustrated in each scatter plot, revealing the capability of the sediment export estimation method used in this study.

The hydrometric data taken at the most downstream gauge of each of the 16 primary rivers spanning from 1970–2010 are used [29] (Fig 1) to compare with the recent study for rainfall-runoff trend detection [24]. All the downstream gauges are close to the coast but beyond tidal influence, giving a fair estimate of sediment discharge to the ocean. The historical runoff and sediment export from the 16 rivers are analyzed and separated into two periods, i.e. early stage (1970–1989) and recent stage (1990–2010), to examine if any changes have occurred in recent years. The mechanisms of sediment transport are also addressed. We hypothesize that the increase of runoff extremes lead to increased sediment export, and that the response is nonlinear/disproportionate due to triggering thresholds and carry-over effects.

## Materials and Methods

### Hydrometric Dataset

The hydrometric data were provided by the Water Resources Agency (WRA) of Taiwan. At each hydrometric gauge, water discharge (Q) was measured daily (or hourly for the typhoon/rainstorm events) and the suspended sediment concentration (Q_c_) was measured at an average frequency of 24 samples per year using a USDH-48 depth-integrating suspended sediment sampler [3]. The suspended sediment discharge (Q_s_) was estimated by multiplying the corresponding Q by Q_c_. The 16 rivers, whose drainage basins range between 350 and 3,250 km^2^, collectively drain 18,700 km^2^, equivalent to half of Taiwan’s total land area. The WRA archive of hydrometric data for some rivers extends as far back as the late 1940s. We used the data starting from 1970 when most of the rivers began to have measurements, except for Houlong River (HLo, 472 km^2^ in watershed area, data started in 1981), Dajia River (DJ, 916 km^2^, 1979), Erren River (ER, 140 km^2^, 1971), and Hoping (HP, 553 km^2^, 1975).

### Calculating Sediment Discharge

Calculating sediment discharge of a river is straightforward if Q and Q_c_ are measured continuously at closely spaced intervals. However, in most cases continuous records of Q_c_ are usually not available, so indirect methods such as sediment rating-curves [29], as in this study, must be utilized. Kao et al. [29] developed stratified time-frame rating curves (i.e. Q_s_ = aQ^b^) and applied them to the 16 Taiwanese rivers. The result successfully reflected yearly and seasonal changes in Q and Q_c_. To develop meaningful rating curves for each time frame, they determined an optimal procedure to separate yearly data into low-flow (November–May) and high-flow (June–October) months and used a FORTRAN program that incorporates limited-range extrapolation in water discharge, representative data points, and meaningful regressions. The separate rating curves in a year were then applied to daily (or hourly for the events) discharges to calculate daily suspended sediment discharge. A bias-correction factor was introduced to reduce residuals. When combined with hourly discharge data, they successfully estimated Q_s_ and Q_c_ in response to episodic events, particularly during typhoons. The calculated sediment discharges by this procedure have been verified by the estimates from the event-based rating curves, the best for estimating sediment discharge in highly variable rivers [29]. The typhoon-triggered sediment hydrograph derived from seasonal rating curves generally agrees closely with that derived from event-based rating curves [30]. Another study has utilized the estimates of the daily discharges to explain the role of lithology, episodic events, and human activities on sediment discharge from small mountainous rivers [25]. The optimized rating curve method used in this study was validated by comparing the estimated Q_s_ to the observed ones on scatter plots (Fig 1). For all the rivers, most data points fall tightly along the 1:1 line, revealing very good capability of this procedure, particularly for the measurements at the higher-end which are most important in land-to-ocean sediment export estimation [25].

## The enhancement of island-wide sediment export

**Fig 2:**
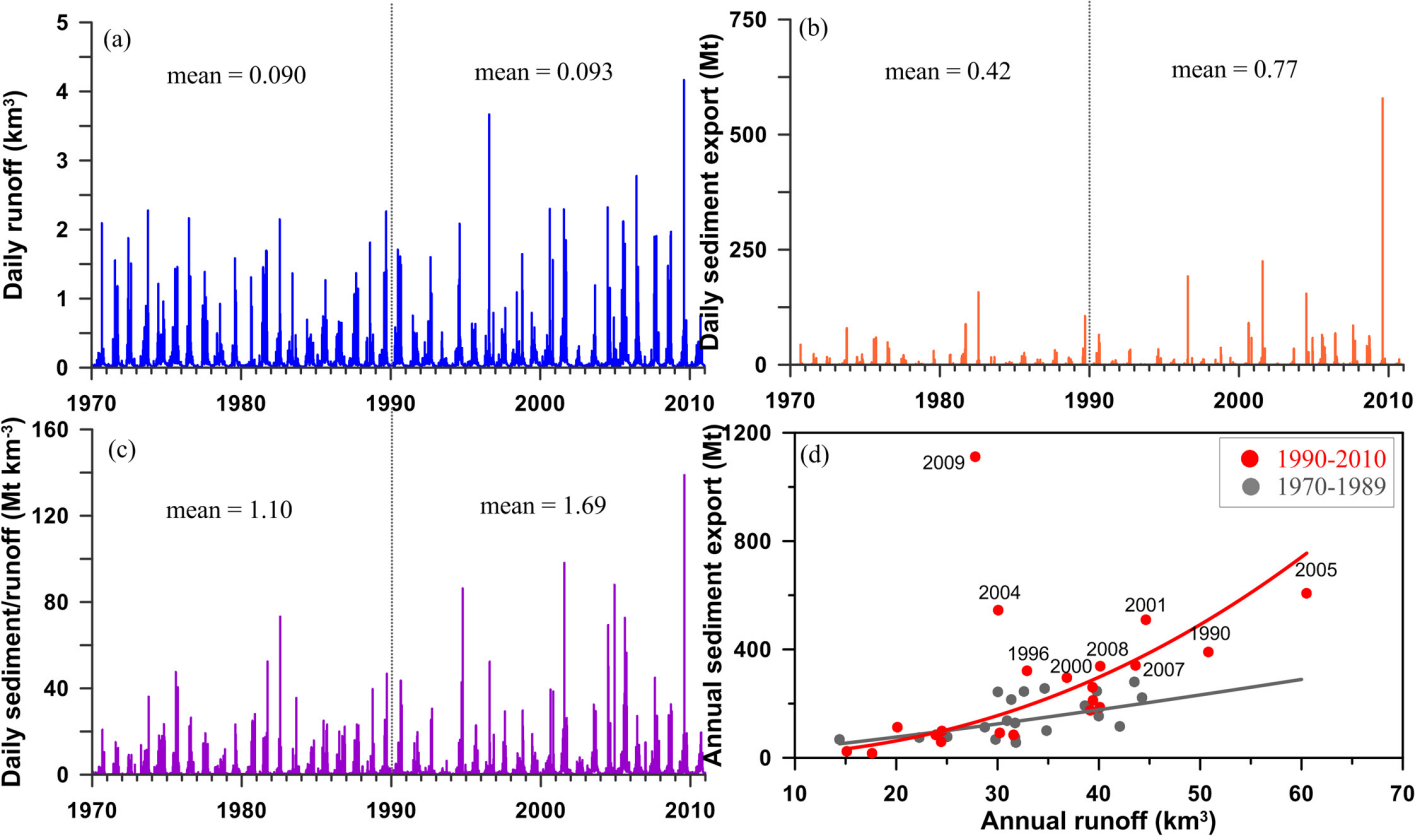
Historical (a) daily runoff [km^3^], (b) daily sediment export [Mt], (c) transport efficiency [Mt km^-3^] defined as daily sediment export divided by daily runoff, and (d) annual sediment export [Mt] against annual runoff [km^3^]. Both runoff and sediment export data are the summation of the 16 rivers.

The time series of historical daily runoff and sediment export from 16 rivers (the summation of the 16 rivers) are illustrated in Fig 2A and 2B, respectively. The patterns of annual cycles are apparent for both runoff and sediment export, peaking in the middle of each year which is mostly associated with episodic events, i.e. typhoons. Intense typhoons, generally lasting for 3–5 days, are often responsible for much of the annual runoff discharge and sediment transport of many Taiwanese rivers [25]. The mean daily runoff in the early stage (1970–1989) was ~0.090±0.024 (variance) km^3^ and then increased slightly to ~0.093±0.032 km^3^ in the recent stage (1990–2010). Although the increment was too insignificant (*p* = 0.149) to pass the Student’s t-test assuming unequal variances, previous studies have demonstrated that the extreme values of runoff are actually significantly increasing [24]. This is due to the increasing rainfall intensity of extreme events [21] and the increasing rainfall amount from typhoons [31], which is not reflected by year-round data analysis. The sediment export is apparently responsive to episodic events as illustrated in Fig 2B [25]. Noticeably, the mean daily sediment export significantly increased (*p <* 0.05) from 0.42±12.5 Mt in the early stage to 0.77±90.85 Mt in the recent stage, revealing a disproportionate increase in sediment export at relatively unchanged daily runoff. As a result, transport efficiency, defined as daily sediment export divided by daily runoff, significantly increased from 1.10±7.43 Mt km^-3^ to 1.69±20.79 Mt km^-3^ (*p < 0*.*05*), indicating more sediment being delivered to the ocean per unit of runoff in the recent stage compared to the early stage (Fig 2C). The significant increases of transport efficiency are found in all the flow regimes, i.e. low-flow months, high-flow months, extremes (defined as when daily runoff is larger than the 97^th^ percentile in a year) and high-flow months excluding the extremes (not shown). Increasing supply of sediment may be anticipated as it is well known that landslide erosion is positively correlated to rainfall intensity and cumulative rainfall amount [23], both showing increasing trends in the past decades [20–21]. The relations between annual island-wide sediment export and runoff in Fig 2D apparently follow two rating curves for the early (grey curve) and recent stages (red), respectively. This shows again that at any given runoff (particularly evident at runoff >30 km^3^) more sediment was exported in the recent stage compared to the early stage.

**Fig 3:**
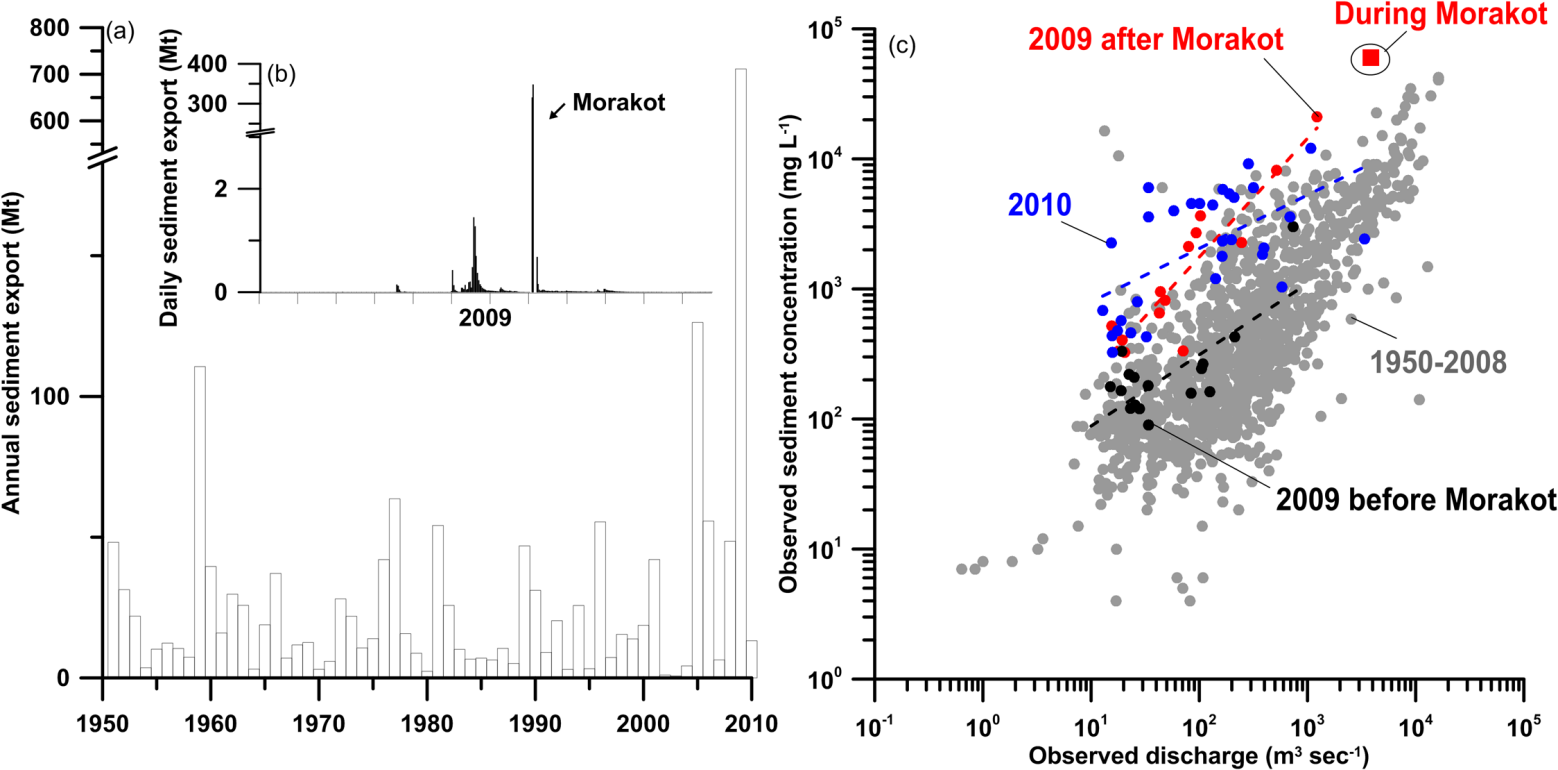
(a) Historical annual sediment export, (b) daily sediment export in 2009, and (c) the historically sampled sediment concentration against the corresponding discharge (the Q_c_-Q relation, gray dots), in Gaoping River. The Q_c_-Q relations for year 2009 (before, during, and after Typhoon Morakot shown as black dots, red square, and red dots, respectively) and 2010 (blue dots) are especially highlighted.

**Fig 4:**
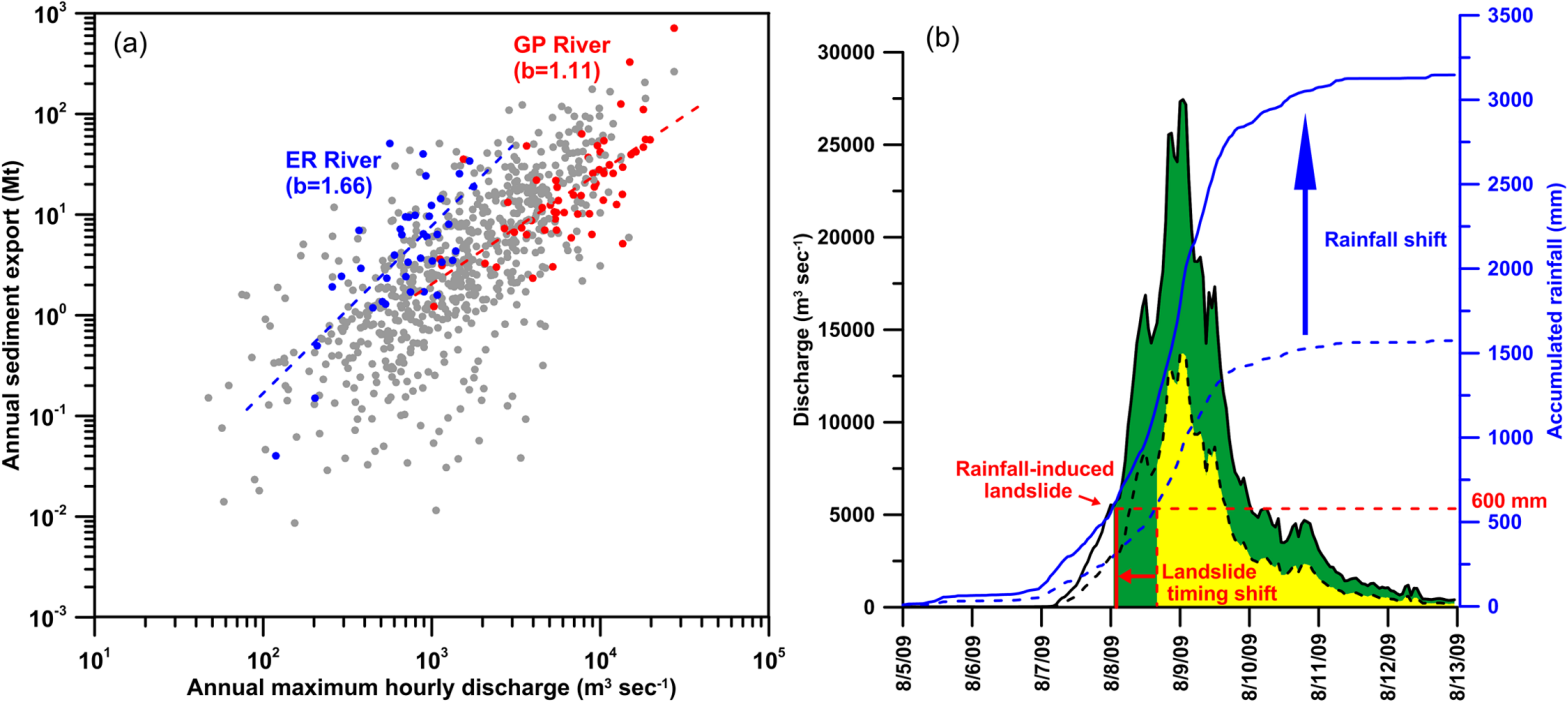
(a) The relation of annual sediment export [Mt] against annual maximum measured hourly discharge [m^3^ sec^-1^] for the 16 rivers (data for Gaoping and Erren Rivers are especially highlighted in red and blue dots, respectively), and (b) a schematic diagram showing the impacts of the increased rainfall intensity, taking typhoon Morakot as an example. The solid blue and black curves represent the observed cumulative rainfall and hydrograph, respectively, for Morakot. The dashed blue and black curves stand for a hypothetic (early stage) event whose rainfall intensity is half of that of Morakot. We assumed that landslides occur at a cumulative rainfall of 600 mm. The left margin of the yellow and green zones represent the time when the river began to transport substantial fluvial sediment in the early stage and during Morakot, respectively. The coefficient b in (a) is the exponent of the power-law relation, i.e. y = ax^b^, between sediment export and maximum hourly discharge.

The red dots, which obviously show higher sediment export at a given runoff (compared to the grey dots in Fig 2D), represent invasions of powerful typhoons, in terms of rainfall intensity/amount, in 1996, 1990, 2000, 2001, 2004, 2005, 2007–2009, among which four of the most catastrophic typhoons occurred after 2001. Although there was the M_w_7.6 Chi-Chi earthquake in 1999, which was followed by a period of enhanced mass wasting and fluvial sediment evacuation mostly in a tributary of Jhoushuei River (JS), the fluvial sediment export was returning progressively to pre-earthquake levels in about six years [32]. Compared with earthquakes, which seldom trigger large landslides unless the magnitude is large, typhoons are indeed a significant landslide trigger [23] and contribute most of the fluvial sediment export in Taiwan [3,20,30]. Without typhoons, earthquake-generated landslides are hardly delivered to the rivers and hence to the ocean. The super typhoon Morakot provides a good example on how sediment is transported in the river. Typhoon Morakot, a devastating typhoon with a record-high rainfall (~1676.5 mm in the first three days, and collectively ~3 m, i.e. approximately the mean annual amount) in southern Taiwan for the past 50 years, ravaged Taiwan from 7^th^ to 11^th^ August 2009 bringing unmeasurable damage and more than 400 casualties [31,33]. The Gaoping watershed (GP in Fig 1), one of the most seriously impacted watersheds by Morakot, experienced 3856 new landslides, covering 116 km^2^ and producing 534 Mm^3^ sediment [23]. The largest landslide occurred on 9^th^ August, having a volume of 25 Mm^3^ (known as Shiaolin landslide, [33]). During this event, Gaoping River discharged ~700 Mt sediment (updated from 450 Mt in [20], see below), which is >90% of the total annual discharge in 2 days (Fig 3B) and ~30× higher than the river’s long-term mean annual load of ~23 Mt (exclusive of Morakot, Fig 3A). There was so much sediment discharged that several submarine landslides and associated sediment-laden turbidity currents formed and caused a succession of submarine cable damage downstream of Gaoping Canyon all the way to the Manila Trench [34–35]. In this case, the water discharge increased by two orders of magnitude (from <1000 to ~27000 m^3^ sec^-1^, black solid line in Fig 4B) within 2 days (a common feature in Taiwan, [30]), and the suspended sediment concentration increased from <10 to ~400 g L^-1^, estimated from the seasonal rating curve with hourly discharge [29]. A previous study reported a conservative value, i.e. 450 Mt, to obey the criteria of rating curve establishment in avoiding excessive extrapolation [29] because the WRA only captured one sediment concentration data point, ~60 g L^-1^, at the discharge of ~3800 m^3^ sec^-1^ during the event (Fig 3C). Therefore, a rating curve with relatively long-term data was used instead, according to the procedure mentioned in Materials and Methods. The updated estimation seems more plausible because the peak discharge during Typhoon Morakot was ~7x larger than the WRA-sampled discharge and a previous study demonstrated >200 g L^-1^ sediment concentration at ~3000 m^3^ sec^-1^ in the JS watershed during Typhoon Mindulle in 2004 [36]. In 2009, Taiwan supplied ~1100 Mt of suspended sediment to the ocean (Fig 2D), which is ~2.8x larger than the long-term average of ~384 Mt yr^-1^ [3]. If we use 450 Mt for the Gaoping export in 2009, it would be ~850 Mt, still the highest value in the record.

To illustrate the sediment supply in the watershed, the sediment response of the river can be explored by considering the relationship between sediment concentration and discharge (Q_c_-Q relation) as shown in Fig 3C. Before Typhoon Morakot, the sediment concentration was relatively low for a given stream discharge (black dashed line in Fig 3C). In the post-typhoon period in 2009, the sediment concentration at a given water discharge was considerably higher due to abundant sediment supply from the landslides, and the slope of the log–log linear regression was apparently steeper (red dashed line). The influence of Morakot remained in 2010 although the regression slope was gentler (blue dashed line). It still represented a higher level of sediment supply at a given stream discharge compared to the historical observations from 1950–2008. Abundant landslide associated sediment remained in the river channel or hillslope [37] and was gradually flushed out in the following events which still supplied more sediment. The fluvial sediment export reflects the interaction between sediment supply and stream power and is still a tangling issue. Similar phenomena can often be found in many Taiwanese rivers [20,30]. The Q_c_-Q relations in Taiwanese rivers migrate back and forth frequently in connection with typhoon strength, earthquakes, and human activities, e.g. road construction and reservoir filling [25,29,32]. Although the sediment supply plays an important role in influencing the regression coefficient and the overall sediment load, the sediment response can be seen to be primarily transport-dominated [29,38] except in watersheds underlain by metamorphic rocks [39]. If typhoon rainfall intensity keeps increasing as currently found [21,31,40] in Taiwan, it is very likely that the future will be characterized by increasing sediment supply and fluvial sediment export.

## The magnified response of sediment export

Episodic events, i.e. typhoons, dominate annual sediment export as shown in Fig 3B. In Taiwan, most sediment erosion and delivery occur in response to typhoon-generated floods, as evidenced by the fact that >75% of the long-term flux occurs in <1% of time [25]. This feature is also demonstrated by a fairly good relationship between the annual sediment export and the recorded annual maximum hourly discharge for each river (Fig 4A). With this relationship, the annual sediment export from a river could be roughly estimated from maximum hourly discharge data although each river follows different relationships depending mainly on the lithology in the watershed [25]. The slope of the regression line for Erren River (blue dashed line in Fig 4A) is steeper than the one for Gaoping River (red dashed line), reflecting the fact that Erren River drains a highly erodible lithology, mostly mudstone. Erren River is the highest sediment-yielding river in Taiwan despite the fact that it drains a low-gradient watershed with relatively low runoff [25]. Besides, erratic sediment supply like the massive landslides triggered by Typhoon Morakot in the Gaoping watershed may deviate the annual sediment export from the regression line (the red dot having the largest y value in Fig 4A). Fig 4A clearly shows that sediment export in Taiwan is basically dominated by event discharge. The estimation of fluvial sediment export can further benefit from good estimations in landslide inventory, together with hourly discharge data [24,39].

Fig 4A illustrates the phenomenon that a power-law relation, i.e. y = ax^b^, exists between the annual sediment export and annual maximum hourly discharge with the exponent b > 1. The annual maximum hourly discharge can be directly related to the event rainfall intensity [41]. Whenever the event rainfall intensity and, hence, the maximum hourly discharge increases, a magnified response of sediment export can be expected according to the relationship in Fig 4A. For example, when the maximum hourly discharge doubles in Gaoping River with b = 1.11, the sediment export increases by ~2.2-fold. When the maximum hourly discharge doubles in Erren River where b = 1.66, the sediment export rises by ~3.2-fold, given the assumption of unlimited sediment supply from the watershed, which is very likely true in most of the rivers in Taiwan. The magnified response of the sediment export is also illustrated in Fig 4B where solid curves are the observed rainfall and hydrograph during Typhoon Morakot and the dashed curves stand for a hypothetical (of early stage) event in which hourly rainfall intensities are assumed to be half of those of Morakot. The hydrograph for the hypothetical event is drawn as half of the observed one simply for illustrative purposes. When the cumulative rainfall in an event reaches the threshold of 600 mm, landslides are likely to occur in Taiwan. When cumulative rainfall is greater than 600 mm, more massive landslides might be further triggered [23], like the Shiaolin landslide mentioned above which occurred at ~1670 mm of cumulative rainfall [33]. In the early stage, landslides that provided abundant sediment to the river did not occur as early as those during Morakot. The left margin of the yellow and green zone in Fig 4B represent the time when the river begins to transport substantial fluvial sediment for the hypothetic lower-intensity event (early stage) and Morakot, respectively. Because the landslide occurrence shifts to an earlier time in the hydrograph, the green zone is more than 2x larger than the yellow zone, explaining again the magnified response of sediment export caused by increased rainfall intensity. The increase of daily sediment to runoff ratio also explains the magnification. In the early stage, 1 km^3^ runoff could transport sediment of 1.10 Mt (Fig 2C). If the ratio remains unchanged, 2x larger runoff will transport 2.20 Mt sediment. However, the 2x larger runoff actually transported 3.38 Mt sediment which is ~3x larger than the amount in the early stage.

Sediment export involves with a succession of processes, such as erosion, transportation, and deposition. Only a small proportion of newly typhoon-triggered landslide material is usually flushed out by the flood generated by the same typhoon. Previous studies stated that the delivery of landslide sediment to channels ranges from 20% to 70% depending primarily on the volume of the landslide, the mobility of the material on the hillslope, the type of mass wasting process, the behavior of sediment at hillslope–channel junctions, and the characteristics of the terrain [42–43]. Moreover, only a small proportion of sediment delivered to channels is further transported to downstream sections [37]. In other words, the sediment mobilized during extreme events can be either transported rapidly downstream while stream power (discharge) is high, or temporarily stored within the watershed and flushed out during later events (carry-over effects). Temporal colluvial terraces or fans are commonly seen spreading along stream channels in Taiwan. Although Gaoping River seems to have discharged ~70% of the Morakot-triggered landslide sediment material (given the bulk density of landslide sediment = 2 Mg m^-3^), the fluvial sediment is actually a mixture of old and new landslide material. Downstream sediment loads reflect a complex response to both newly delivered and stored sediment supply and ambient hydraulic conditions. Accurate sediment transport estimation cannot be achieved simply from known water discharge time series using a sediment rating curve, but requires detailed knowledge of the spatial and temporal patterns of the hillslope mass wasting and sediment transfer into the fluvial system [39]. However, it is reaffirmed here that the transport capacity, rather than supply, dominates the annual sediment discharge for most of the rivers in Taiwan [29,38].

## The chain reactions from increased rainfall intensity

Taiwan is facing a changing rainfall pattern, a trend where lighter rain is descending and heavier rain is ascending [40,44], which is strongly related to global warming. The top 10% bin of rainfall intensity, mostly from typhoons, has increased by about 95% for each degree Kelvin increase in global mean temperature [21]. Besides, the pattern of typhoons invading Taiwan has also been changing. An abrupt shift in the number of typhoons influencing Taiwan has been found from 3.3 typhoons per year (1970–1999) to 5.7 per year (2000–2006), resulting from the warm sea surface temperature anomalies over the equatorial western and central Pacific [45]. Forceful typhoons (category 4 and 5) have tended to occur more frequently in May since the year 2000. Before 2000, intense typhoons occurred in May around once per decade, but now almost once per year [46]. Moreover, it has also been found that typhoons tend to translate more slowly, which partly explains the increasing rainfall intensity of the extreme rainfall events [31]. A previous study has calculated the relative changes of extreme rainfall events (99.0–99.9 percentiles) from 1971–1990 to 1991–2010 using data from island-wide rain gauges in Taiwan and found an average increase of 22.6% [24]. The changing rainfall pattern is challenging Taiwan’s government in dealing with water resource management and disaster prevention. It is important to note that rainfall intensification is not only happening in Taiwan but generally in the ‘wet’ region (e.g., low latitude and the West Pacific region) [47].

**Fig 5:**
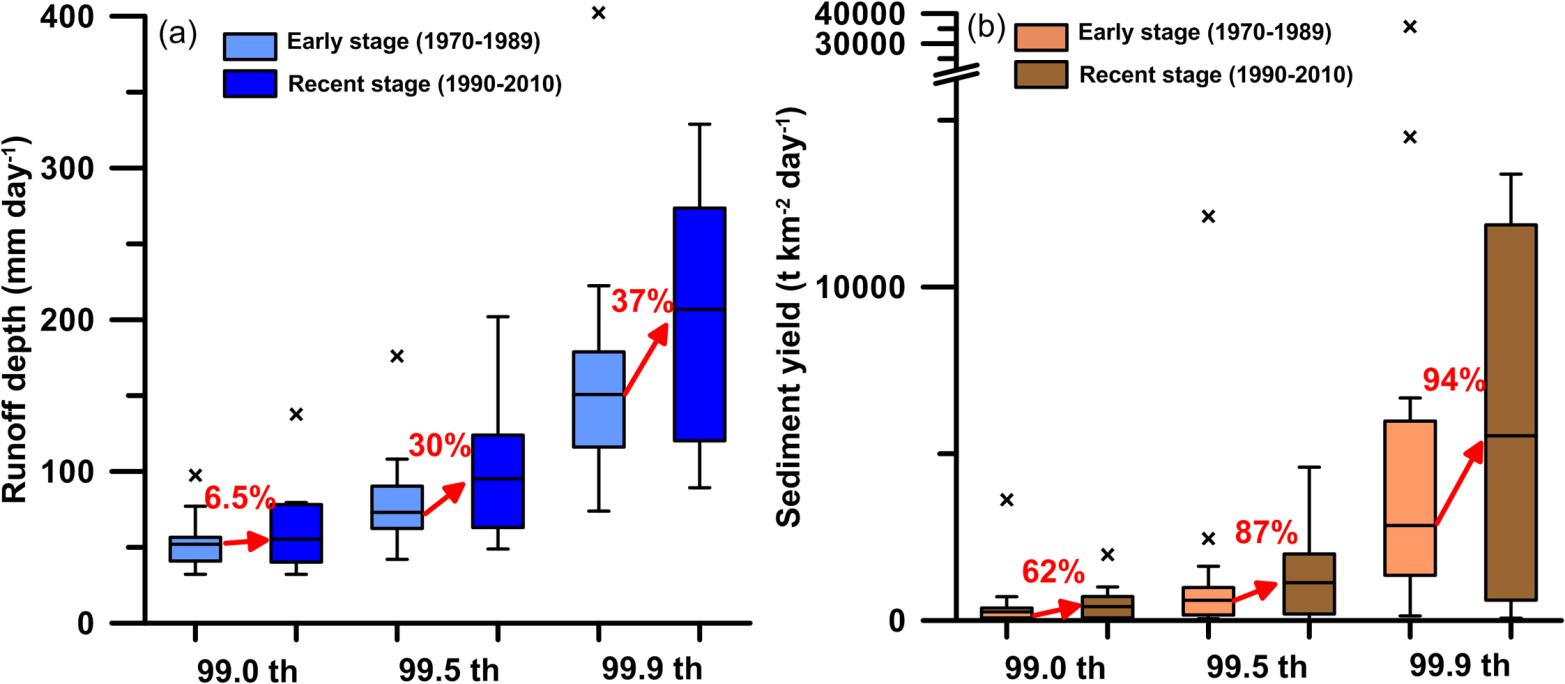
The changes of the 99.0-, 99.5-, and 99.9-percentile of (a) runoff depth and (b) sediment yield between the early stage (1970–1989, shown in lighter blue and brown boxes) and the recent stage (1990–2010, shown in darker blue and brown boxes). Each box-whisker contains values from the 16 rivers. Both runoff and sediment yield extremes have increased in the recent stage compared to the early stage.

Runoff response to the intensified rainfall is not necessarily linear depending on the status of the water storage in the watershed [20]. A watershed acts like a sponge absorbing rainfall and yielding runoff. However, the water yield, defined as the ratio of runoff to rainfall, changes with the rainfall intensity. A previous study found that approximately 60% of the rainfall converts to runoff when the daily rainfall is <75 mm day^-1^ for watersheds with little human disturbance in Taiwan (e.g. no major water supply devices and land use change) [24]. With the water storage being occupied by increased rainfall, almost all excess rainfall becomes runoff, resulting in ~100% of water yield. This pattern leads to stronger magnitude of increase in runoff than rainfall itself. In the above-mentioned study [24], the observed relative changes (between 1971–1990 and 1991–2010) of runoff extremes (99.0–99.9 percentiles) are between 27.4% and 62.2%. We also examined the relative changes of runoff extremes between the early and recent stage for the 16 rivers in this study. To remove the size effects among the watersheds, normalized runoff depths are illustrated in Fig 5A. The values of extreme runoff depths (99.0-, 99.5-, and 99.9-percentile) of the 16 rivers are generally higher in the recent stage compared to the early stage (exclusive of the outliers in the early stage). All the outliers are from Erren River where some severe rainfall events happened in the early stage. Compared with the early stage, the medians of the 16 rivers in the recent stage increased by 6.5%, 30%, and 37% for the three percentiles, respectively. These increases are not as high as those reported in the previous study [24], because our runoff gauges are located further downstream. Larger watershed size and human manipulation have very likely attenuated the fluctuations.

We further examined the relative changes of extremes in sediment export between the early and recent stage for the 16 rivers. The extremes of sediment yields, sediment export normalized by watershed area, are illustrated in Fig 5B. Compared with the early stage, the medians of the 16 rivers increased by 62%, 87%, and 94% in the recent stage for the three percentiles, respectively, which is much higher than that of the runoff and rainfall extremes. It results from the flushing nature of the fluvial sediment export in Taiwanese rivers [29], given the unlimited supply of sediment in the watershed from either the fresh sediment of newly-generated landslides or the old sediment deposited in the foothills and interfluves [37,39]. Landslides in Taiwan commonly occur in areas where the slope is steeper than 20° [22], and the probability of reactivation of old landslides is >50%, leading to carry-over effects with mass wasting continuing until the slope of the scar reaches a stable condition. In our study, we could have possibly underestimated the increases in sediment export because of human facilities, like upstream dams and reservoirs which usually trap substantial amount of sediment and hence mitigate the climate-driven increases. Hence, an even stronger increase can be expected after removing the effects of sediment trapping.

Sediment associated damages/hazards deserve extensive attention in Taiwan. The siltation of upstream reservoirs is a serious problem, not to mention the destructive power of the turbid flood. Roads, bridges, and houses destroyed by flood are common in almost every typhoon event every year [20]. Fluvial sediment export is also a recognized conveyor delivering terrestrial material to the ocean [19,36,48]. SMRs in Oceania are well-known hot spots of terrestrial export, having potential biogeochemical significance since many of these rivers discharge onto narrow shelves, facilitating sediment transfer to the deep sea and thereby enhancing the chance for organic carbon sequestration [1,15,19,49]. Landslides can scrape forest biomass and soil from hillslopes [50] and mobilize modern organic carbon recently fixed from atmospheric carbon dioxide via photosynthesis. Fluvial sediment transport may further deliver the modern organic carbon to the seabed via hyperpycnal flow, resulting in long-term sequestration of modern organic carbon [10,30,51]. Given the fact that ~40% of the annual global land-to-ocean sediment export is from Oceania rivers [1], Oceania rivers clearly play an important role in the global carbon cycle. Besides, a recent study also demonstrated that short-lived and intense erosional events associated with efficient sediment transport, such as typhoons, could trigger shallow seismicity or promote the rupture of deep continental earthquakes up to the surface [8]. The chain reactions from increasing rainfall intensity, particularly the magnified response of sediment export, have been found in observations and predicted to be enhanced further in the future. If the globe keeps warming, rainfall extremes which have been proved temperature dependent [21], will very likely intensify to trigger more runoff, sediment, and even seismic-associated hazards.

## Remarks

The 21^st^ century began with numerous unprecedented climatic extremes worldwide. Among them, the signal of rainfall intensification is more and more evident. In this study, the chain reactions from rainfall extreme to fluvial sediment export were investigated in Taiwan, which is characterized by abundant precipitation and massive mass wasting. Our analysis reveals that the island-wide daily runoff remains statistically unchanged, whereas the average daily sediment discharge increases significantly from 0.42 Mt in the early stage (1970–1989) to 0.77 Mt in the recent stage (1990–2010). Hence, stream transport efficiency (represented by daily sediment/runoff) increased from 1.10 to 1.69 Mt km^-3^ for the two stages, respectively. This is likely due to the exceedance of triggering thresholds as well as carry-over effects from previous events. Our results also highlight an increase of sediment supply from watersheds. In landslide-dominated regions, sediment supply is usually not a limiting factor. A previous study demonstrated that the landslide-associated erosion depth increased by ~2-order in magnitude in three reservoir watersheds in Taiwan when the average rainfall intensity of typhoon increased by 2-fold [52], implying a dominant ruling of nature (weather and lithology) that overwhelms the impacts of human activities. In fact, Taiwan government has been spending huge money on maintaining hillslope stability. Landslides occur not only in human-altered areas but also (much more) in the pristine regions. This seems Taiwan’s destiny having such a high rainfall intensity and highly erodible lithology. Though there is a Chinese saying, “Man always conquers Nature”, we are afraid that even the state-of-the-art engineering is still no rival for nature.

In terms of annual total sediment export, the 16 rivers discharged ~154 Mt yr^-1^ and ~280 Mt yr^-1^ of suspended sediment (~8,231 t km^-2^ yr^-1^ and ~14,965 t km^-2^ yr^-1^) in the early and recent stage, respectively. Taiwan Island annually exports ~538 Mt suspended sediment to the ocean if the sediment yield for the recent stage is applied to the whole island. The increasing sediment export can be linked to climate extremes. The medians of the runoff depth extremes (99.0–99.9 percentiles) among the 16 rivers have increased by 6.5%-37%. More importantly, the medians of the sediment yield extremes (99.0–99.9 percentiles) have increased by 62%-94%, revealing the non-linearity in this chain reaction. The importance of sediment export has been well recognized in regard to hazard mitigation and carbon cycling. Facing the tendency towards magnified sediment discharge, we will have to consider lowering human activities on unstable terraces, fans, and flood plains to avoid increasing hazards and damage. The magnified sediment discharge carrying more carbon (biomass, soil, and rock-derived) from land to ocean plays an increased role in the carbon cycle. Our observations show that the transfer of carbon might have been speeding up, which is crucial in understanding the carbon budget in the watersheds and beyond. The chain reactions observed in this study should be carefully explored in other regions for hazard prevention and to improve our knowledge on sediment and carbon budgets.

## Supporting Information

S1 Table (A). Historical daily runoff [km^3^], daily sediment export [Mt], transport efficiency [Mt km^-3^] defined as daily sediment export divided by daily runoff. The table shows the summation of the 16 study rivers. The table can be used to make Fig 2A, 2B and 2C. (B). Annual sediment export [Mt] against annual runoff [km^3^] off the 16 rivers. The table can be used to make Fig 2D.(C). Annual sediment export [Mt] and annual maximum measured hourly discharge [m^3^ sec^-1^] for the 16 rivers. The table can be used to make Fig 4A. (XLSX)
